# Plasma immobilization of azobenzene dye on polyamide 6 polymer

**DOI:** 10.1038/s41598-023-27484-9

**Published:** 2023-01-18

**Authors:** Mohammad Reza Yari, Mohammad Sadegh Zakerhamidi, Hamid Ghomi

**Affiliations:** 1 Laser and Plasma Research Institute, Shahid Beheshti University, Tehran, Iran; 2 Faculty of Physics, University of Tabriz, Tabriz, Iran; 3 Research Institute for Applied Physics and Astronomy, University of Tabriz, Tabriz, Iran; 4 Photonics Center of Excellence, University of Tabriz, Tabriz, Iran

**Keywords:** Atomic and molecular collision processes, Macromolecules and clusters, Plasma physics, Other photonics, Nanoscale devices, Polymer chemistry, Surface chemistry

## Abstract

Plasma treatment of polymeric materials is a cost-effective and efficient technique to modify the surface and change the constituent unit configuration. This research investigates the effects of argon DC glow discharge plasma on pure and DR1 dye-loaded polyamide 6 polymer films and stabilization of dye on the surface. Plasma breaks some bonds and activates the surface through creating reactive structures such as free radical sites on the surface and increases tertiary amides on the surface of polymer. Besides, this process alters surface topographical characteristics and conformation of azobenzene dye which are effective on the durability of the dye on the surface. Plasma causes interactions of the dye with the polymer and immobilizes the dye on the polymer. On the other hand, these interactions lead to changes in the dye's optical and geometric isomeric activity and stability. This work studies the chemical and morphological changes of polyamide 6 by plasma with AFM and spectroscopic methods. Furthermore, the aging of nylon 6 films loaded with DR1 dye is measured, and the conformational changes of the dye are investigated. Plasma stabilizes the dye on the polymer surface through making changes of chemical and physical properties on the surface components.

## Introduction

Polymers have a variety of applications due to their different physical and chemical properties^1^. Polyamides are the first types of globally mass-produced thermoplastic engineering plastics, produced commercially in two forms of films and fibers in the industry^2^. Polyamide 6, as a type of polyamide, is a significant polymer in the industry and is classified in the category of semicrystalline polymers^3^. This polymer is a synthetic polymer extensively used in electronic devices, machine industry, and military equipment^4,5^. Polyamide 6 has favorable properties, such as suitable chemical and aging resistance^6^, appropriate electrical and thermal resistance^7^, excellent mechanical properties, and low contact friction^8^. It has both hydrophilic parts resulting from carbonyl and amide groups and hydrophobic parts due to the presence of ethylene sequence in its structure^9^.

In general, polymers usually have low surface energy because of the deficiency of polar groups in their structure, leading to low wettability and adhesion^10^. In these materials, surface properties sometimes limit their uses in different applications. As a result, improving the surface properties of polymers has great importance in applications, such as coating or printing^11^. There are different methods of surface modification for changing the surface properties while maintaining the bulk profile of material, like chemical modification^12^, radiation^13^, and thermal treatment^14^, which is crucial for various applications. Disadvantages of the mentioned methods include using hazardous chemicals, high energy consumption, and expensive materials and equipment.

Therefore, methods including not using chemical material for surface treatment and modification are more popular and practical nowadays. Plasma treatment of material is an environmentally friendly, clean, dry, chemical-free, and economical method to alter the surface properties^15–17^. Cold plasma is a quasi-neutral medium in which particles are in a thermal non-equilibrium state, and heavier particles possess lower temperatures than electrons^18^. The cold plasma treatment of materials is a suitable method to alter the surface characteristics of a polymer without any damage to its bulk^19,20^. A variety of energetic particles in plasma media, including ions, electrons, radicals, metastable species, and photons in the energy range of ultraviolet radiation, affect the surface^21,22^. The depth of modification via the plasma technique for the surface is from the order of 10 nm, without influencing the bulk characteristics^21^. The interactions between the plasma and the polymer surface lead to results, such as chain scission^23^, etching, polymerization, formation of new covalent bonding and functional groups, crosslinking^24^, generation of polar groups^25^, and elimination of surface contamination^26^. Thus, the plasma can change the chemical, physical, mechanical, and adhesion properties of the surface of polymers^27,28^. In addition, the plasma affects properties, such as printability, wettability, and biocompatibility of the surface^29^.

Dyeing of materials is done in different ways, and dyeing after manufacture is one of the economical methods to reduce the consumption of dyes. But the main problem in this method is low color stability on the surface due to environmental stress conditions like heat, sunlight, and humidity^30^. Dye immobilization on the surface of material is an important subject that depends on surface physical and chemical properties^31,32^. Disperse Red 1 (DR1) dye is a pseudo-stilbene type azobenzene with push–pull characteristic through its electron donor and acceptor substituents^33^. Azobenzene is a molecular switching system with two phenyl rings linked by (–N=N–) that provides reversible photoisomerization under ultraviolet light irradiation^34^. Azobenzene molecules are found in two configurations of E (trans) and Z (cis) isomers^35^. More stable trans isomer converts to cis conformation during exposure to UV light^36^. The molecule is transferred to cis state with about 50 kJ mol^−1^ more energy, and the cis isomer changes to trans after thermal relaxation^37^. Photoisomerization of trans azobenzene causes geometrical structure change and brings the aromatic rings closer to each other^38^. Azobenzenes have a wide range of applications, such as optical data storage^39^, sensors^40^, nonlinear optics^41^, nanomachines^42^, drug delivery, holographic^43^, optical and solvatochromic applications, and so on^44,45^, due to their photoswitching, molecular motion, electronic, and optical properties. As a result, the dye must be immobilized on the surface of material for better efficiency in various applications^40^. Also, polymers containing azo dyes have extensive optical applications through their low cost and desirable optical response and have the potential to be used in optoelectronic devices^46,47^.

In this work, pure and dye-loaded polymer films were exposed to glow discharge plasma to apply plasma-induced changes on the surface of prepared samples. Fourier-transform infrared spectroscopy (FT-IR) and atomic force microscopy (AFM) were employed to evaluate the chemical and morphological changes of the polymers’ surface as the result of plasma treatment. The accelerated aging process was used to estimate dye fastness on the surface of the polymer samples. UV–Vis spectroscopy was applied to measure and compare the effects of plasma on dye fastness of the polymer films and dye conformational composition before and after plasma treatment.

## Results

### Plasma effect on pure and DR1 doped nylon 6 polymer

**Figure 1:**
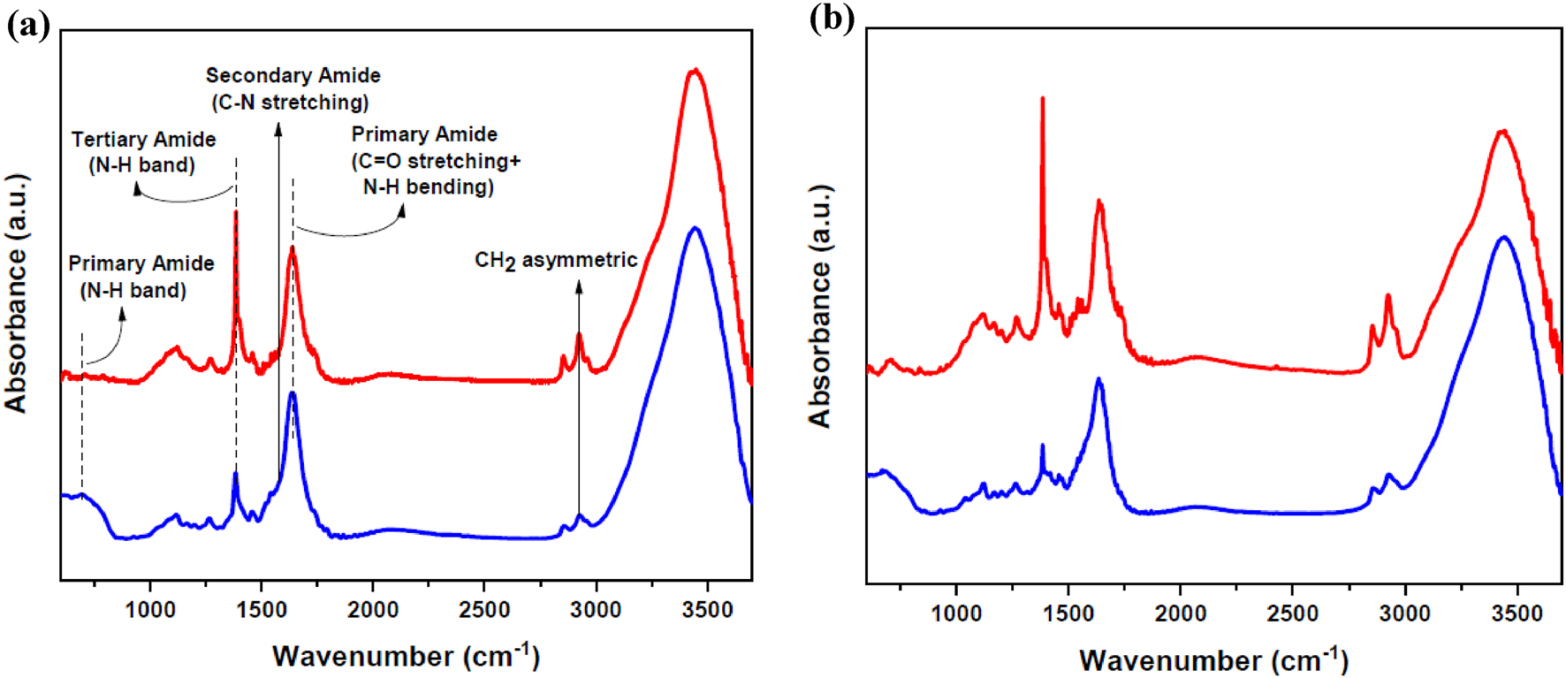
FT-IR spectra of (**a**) pure polyamide 6 polymer (**b**) DR1 dye-doped polymer and (blue lines) untreated (red lines) plasma-treated samples with argon gas for 300 s modification.

After preparing the thin films of pure and DR1 dye-doped polyamide 6, each sample was placed in the plasma reactor for treatment with the argon plasma. FT-IR spectra of the thin layer samples show that the argon plasma treatment of the films, for 300 s, changes the chemical structure of thin polymeric layers. Given that the glow discharge plasma affects the surface, it can be said that these chemical changes have occurred on the surface of polyamide 6. Figure 1 shows the changes of the bonds in the FT-IR spectra of the pristine and the plasma-treated samples. The more detailed peaks information of FT-IR spectra of DR1 dye-doped polyamide 6 polymer in Fig. 1b, before and after plasma treatment for 300 s, were presented in Supplementary Fig. S1. Also, Supplementary Fig. S2 shows the spectra of pure and DR1 dye-doped polyamide 6 polymer before and after plasma treatment for 150 s.

The FT-IR spectra show that the absorption bands in the range of 2850–3000 cm^−1^ belong to C-H (CH_2_) symmetric and asymmetric stretching vibrations. The amide group's presence and nitrogen non-bonding electrons conjugation with carbonyl compound in the amide group lead to the reduction of absorption frequency and wavenumber in the carbonyl band because of the resonance effect^48^. So, the nearby peaks at 1635 cm^−1^ are related to the primary amide by overlapping of C=O stretching bands and N–H bending vibrations. The peak located at 1578 cm^−1^ refers to the secondary amide in the polymer structure, and the peak at 1457 cm^−1^ is attributed to the other forms of N–H vibrations. In addition, the observed band in 1383 cm^−1^ corresponds to the tertiary amide group. The wide band in 699 cm^−1^ is ascribed to N–H wagging vibrations.

**Figure 2:**
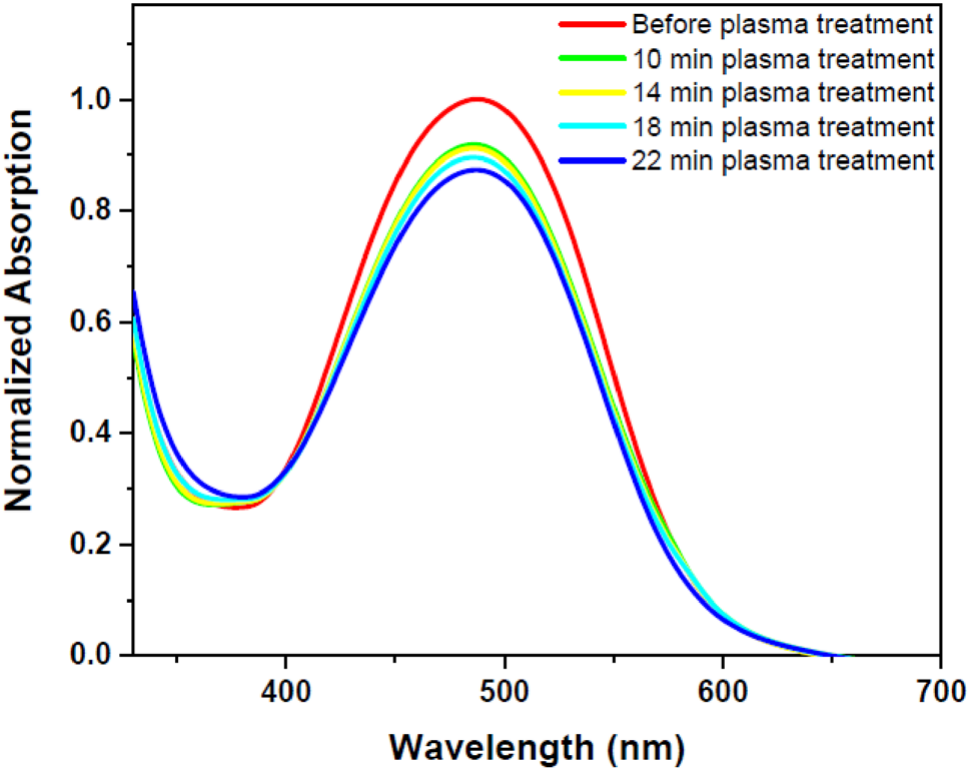
UV–Vis spectra of pristine and argon plasma-treated DR1 dye-doped polyamide 6 polymer film for various treatment times.

**Figure 3:**
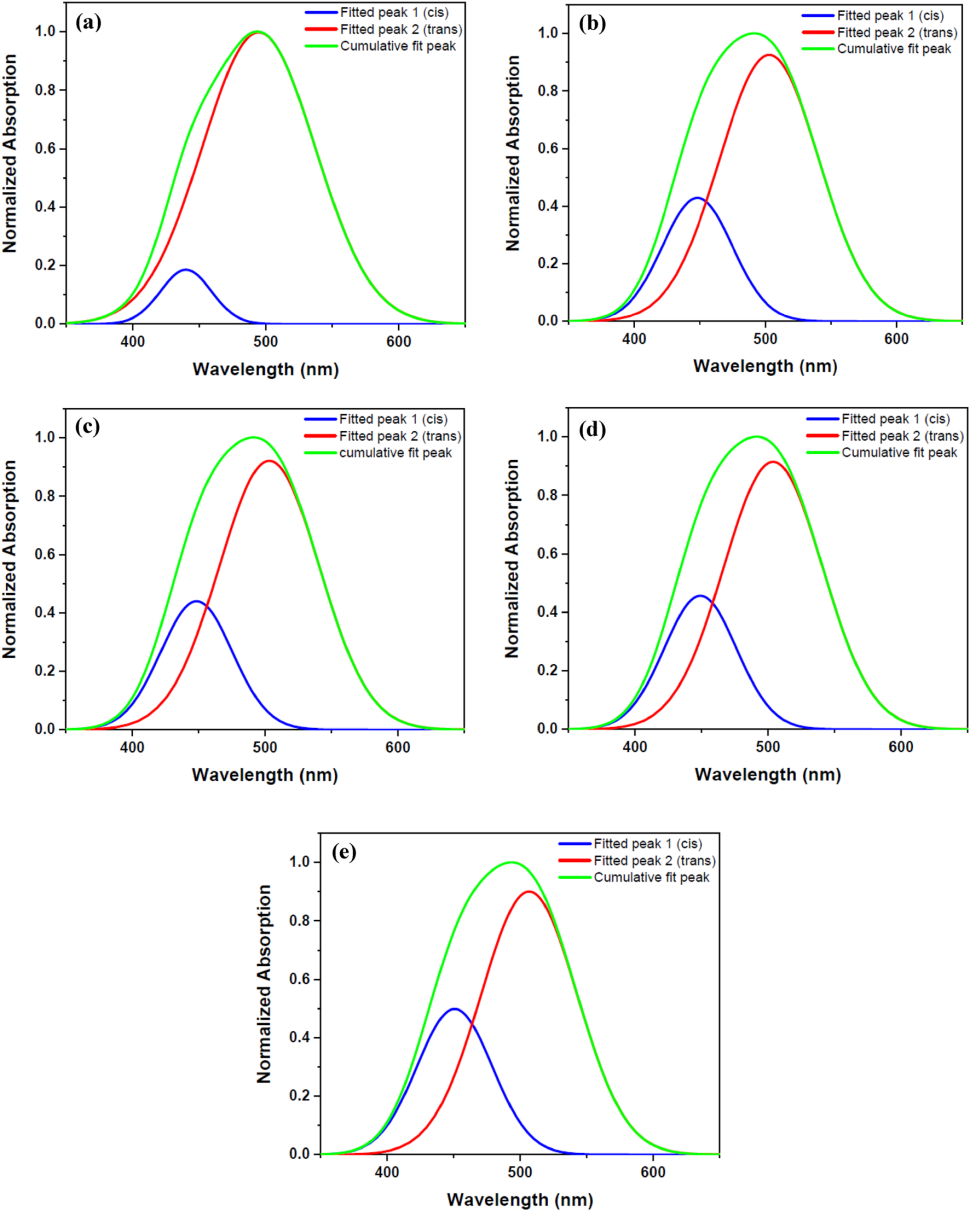
Double peaks fitted for the UV–Vis normalized absorption spectra of DR1 dye-doped polyamide 6 polymer film before and after plasma modification for different treatment times: (**a**) before plasma treatment (**b**) 10 min (**c**) 14 min (**d**) 18 min (**e**) 22 min plasma treatment.

DR1 dye-doped polyamide 6 polymer thin film was exposed to plasma for different treatment times to further investigate plasma effects. Then UV–Vis spectra of the sample for each time interval of plasma treatment were recorded and compared with the sample without plasma exposure. Figure 2 shows the UV–Vis spectra of the untreated and treated sample for different plasma treatment times. Figure 3 reveals deconvoluted double peaks fitted for each UV–Vis normalized absorption spectrum in Fig. 2 assigned to overlapped trans and cis isomeric states of the dye. Table 1 demonstrates the area under each fitted peak, which represents the population of species.

**Table 1:** The amounts of DR1 dye isomeric species and their ratios calculated from fitted double peaks before and after plasma treatment at different modification time intervals for dye-doped polyamide 6.

Samples	S_trans_	S_cis_	S_cis_/S_trans_	S_cis_/S_total_(%)
Before plasma treatment	76.50	6.38	0.08	7.69
10 min plasma treatment	49.23	16.10	0.32	24.64
14 min plasma treatment	48.46	16.57	0.34	25.48
18 min plasma treatment	46.23	16.88	0.36	26.74
22 min plasma treatment	42.23	18.24	0.43	30.16

*S*_*trans*_ area under the fitted peak 1, *S*_*cis*_ area under the right fitted peak 2, *S*_*total*_ area under the cumulative fit peak.

Changes in the chemical structure of the surface and the presence of different materials with polar properties cause changes in the absorption wavelength by the dye’s species. The obtained data from the deconvoluted lines demonstrates that the plasma environment converts several trans isomers to cis form. As can be seen from Table 1, the ratio of cis to the trans population for dye-doped polymer film is 0.08 before the plasma treatment of the sample. Furthermore, after 22 min of plasma treatment, these values increase to 0.43 for the dye-doped polymer. In other words, 7.69% of the total population is related to the isomeric state of cis for the dye-doped polymer sample before the plasma. After 22 min of plasma treatment, the population of cis in the sample reaches the value of 30.16%.

**Figure 4:**
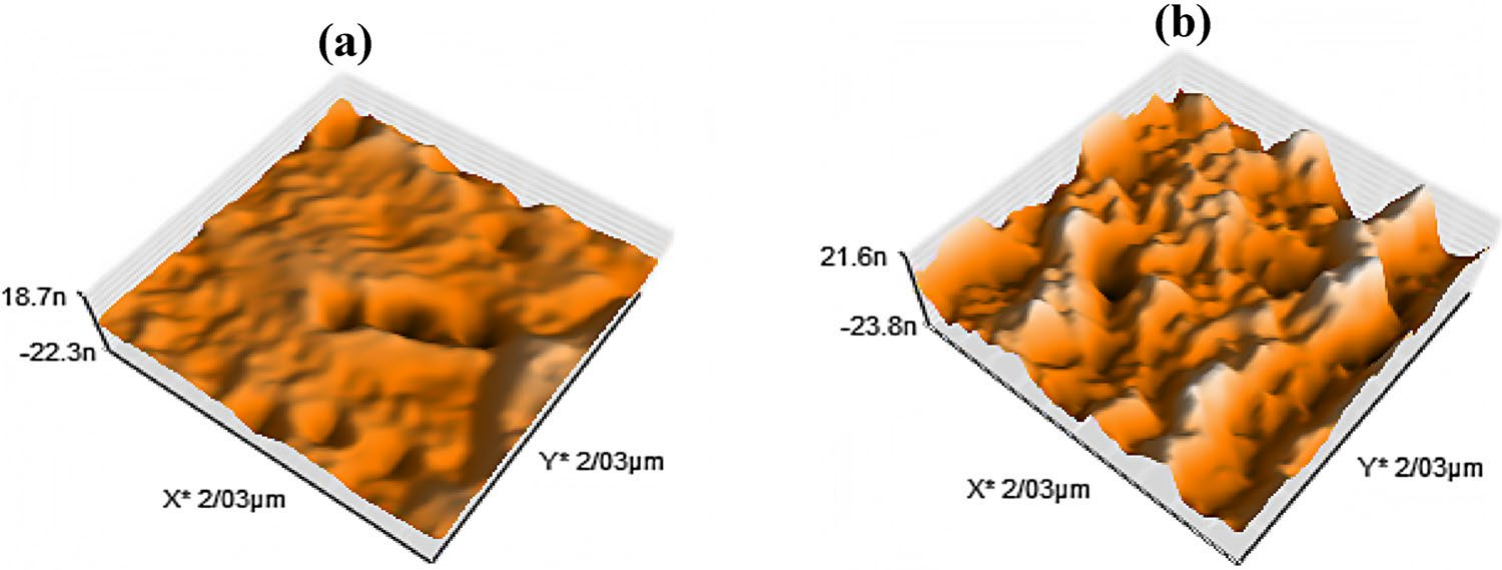
AFM images of (**a**) untreated (**b**) argon plasma-treated DR1 dye-doped polyamide 6 surface for 300 s modification.

Due to the surface treatment nature of cold discharge plasma, it seems that the study of the changes induced by plasma on the surface of DR1 dye-doped polyamide 6 polymer can provide interesting information. An atomic force microscope (AFM) was used to analyze the surface topography of the dye-doped polymer films. The results obtained from the AFM in Fig. 4 show 3D topographical images and the changes of surface roughness of DR1 dye-doped polyamide 6 polymer film by plasma. The comparison between the pristine and the argon plasma-treated sample’s surface for 300 s modification, demonstrates apparent alteration of surface roughness parameters, as presented in Table 2. Figure S3 in supplementary information shows surface roughness before and after plasma treatment for 150 s.

**Table 2:** Comparative presentation of roughness parameters (R_a_, R_q_, and R_z_) of dye-doped PMMA polymer surfaces before and after argon glow discharge plasma treatment for 300 s.

Samples	Roughness parameters		
	R_a_	R_q_	R_z_
Untreated DR1 dye-doped polyamide 6	5.436 nm	6.707 nm	29.353 nm
Plasma-treated DR1 dye-doped polyamide 6	16.412 nm	19.502 nm	64.654 nm

The roughness parameters include the arithmetic averages of the assessed values (R_a_), the root mean square average of height deviations from the mean line (R_q_), and the maximum peak to valley height (R_z_).

### Plasma effect on surface dyeing of DR1 doped nylon 6 polymer

**Figure 5:**
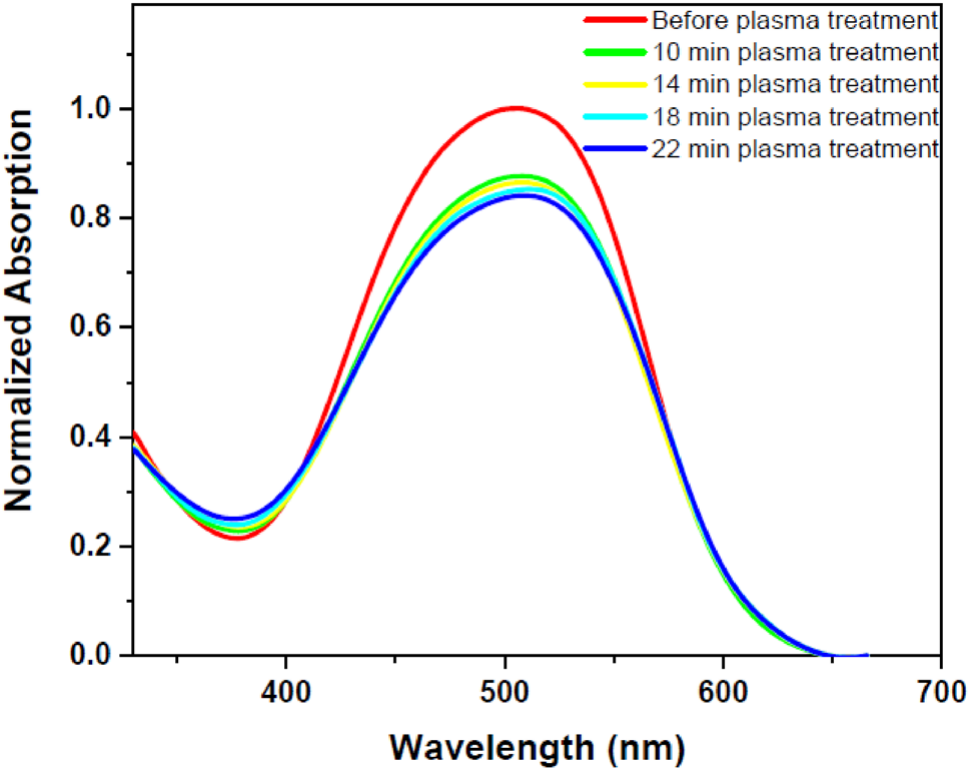
UV–Vis spectra of pristine and DR1 dye-loaded polyamide 6 polymer film with extra adsorbed surface dye through dipping in DR1 dye solution and treated with argon plasma at various treatment times.

**Figure 6:**
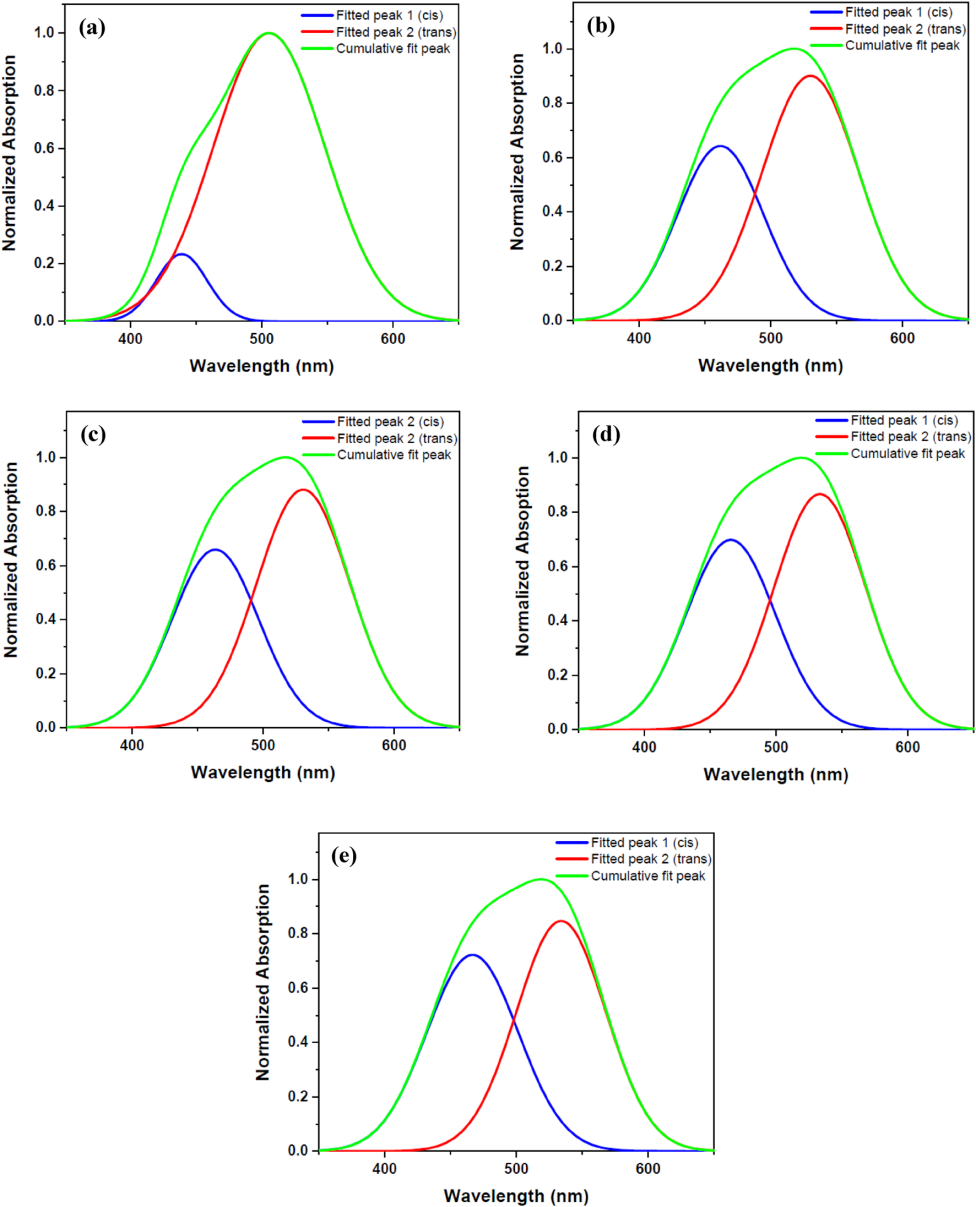
Double peaks fitted for the UV–Vis normalized absorption spectra of DR1 dye doped polyamide 6 polymer film with extra surface dye before and after plasma for different treatment times: (**a**) before plasma treatment (**b**) 10 min (**c**) 14 min (**d**) 18 min (**e**) 22 min plasma treatment.

In this part, DR1 dye-doped polymer film was dipped in DR1 dye solution (in Ethanol solvent 2 × 10^–3^ M) to adsorb more dye on the surface of the sample. The sample was exposed to plasma for various time periods to study and compare the plasma influence on the surface components. UV–Vis spectra were performed for the sample for each time interval of plasma treatment and compared with the state before plasma exposure to investigate the other effects of plasma. Figure 5 shows the UV–Vis spectra of the untreated and treated samples for different plasma treatment times. Figure 6 reveals deconvoluted double peaks fitted for each UV–Vis normalized absorption spectrum in Fig. 5 assigned to overlapped trans and cis isomeric states of the dye. Table 3 demonstrates the area under each fitted peak, which represents the population of species.

**Table 3:** The amounts of DR1 dye isomeric species and their ratios calculated from fitted double peaks before and after plasma treatment at different modification time intervals for dye-doped polyamide 6 with extra surface dye.

Samples	S_trans_	S_cis_	S_cis_/S_trans_	S_cis_/S_total_(%)
Before plasma treatment	38.02	4.06	0.10	9.64
10 min plasma treatment	26.31	16.74	0.63	38.88
14 min plasma treatment	23.40	16.35	0.69	41.13
18 min plasma treatment	21.81	17.24	0.79	44.14
22 min plasma treatment	20.15	17.57	0.87	46.58

*S*_*trans*_ area under the fitted peak 1, *S*_*cis*_ area under the right fitted peak 2, *S*_*total*_ area under the cumulative fit peak.

### Plasma effect on aging of DR1 dye loaded polyamide 6 polymer

The accelerated aging process was used to estimate the persistence of dye against the factors such as heat, UV radiation, and humidity to evaluate the dye immobilization on the surface of the polymer under laboratory conditions. The aging process affects the surface of dye-loaded polymeric samples by different reactions such as surface chemical alteration, oxidation, weak link removal, reorientation of the surface contents, photo-degradation, and reduction of surface energy and activity^49,50^.

**Figure 7:**
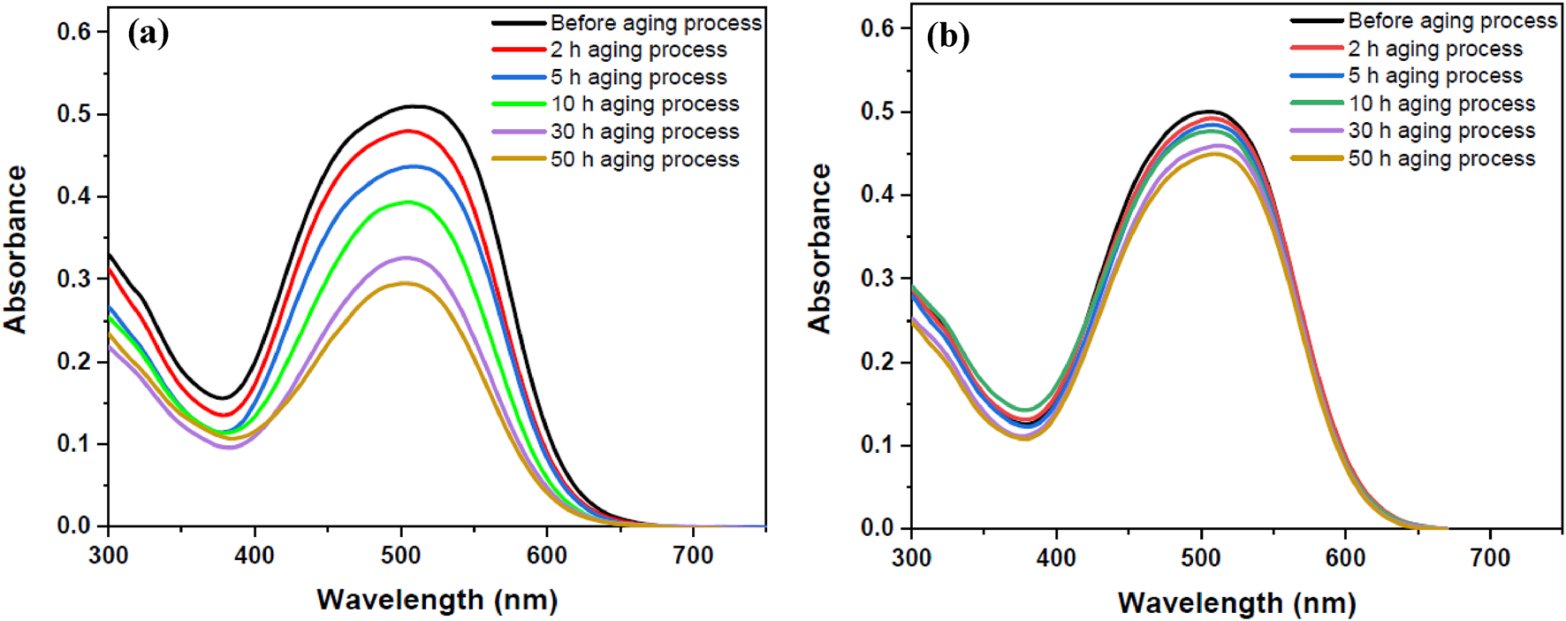
UV–Vis spectra of DR1 dye-loaded polyamide 6 polymer films during different times of aging process: (**a**) untreated (**b**) plasma modified dye-loaded film after treatment time of 300 s.

The plasma treatment time was optimized for dye fastness on the surface of the sample according to the plasma characteristics. For this experiment, dye-loaded polyamide 6 polymer film was exposed to plasma for optimized time of 300 s. The dye was loaded by immersing the pure polymer films in the dye solution before plasma treatment. The samples were washed in ethanol after plasma modification and before exposure to the aging test. The results of aging process were compared with the control sample without plasma treatment under equal conditions. It should be noted that in this part of the experiment, all samples, after the aging process, were stored in a dark environment at a temperature of 10 °C for 24 h to neutralize and relax their temporary effects. The degree of dye fastness on the polymer surface due to the aging process for the untreated and plasma-treated dye loaded samples was determined by UV–Vis spectroscopy. The durability of the dye on the polymer surface is shown in Fig. 7.

### Effect of plasma on color strength of dye-loaded polymeric films

**Figure 8:**
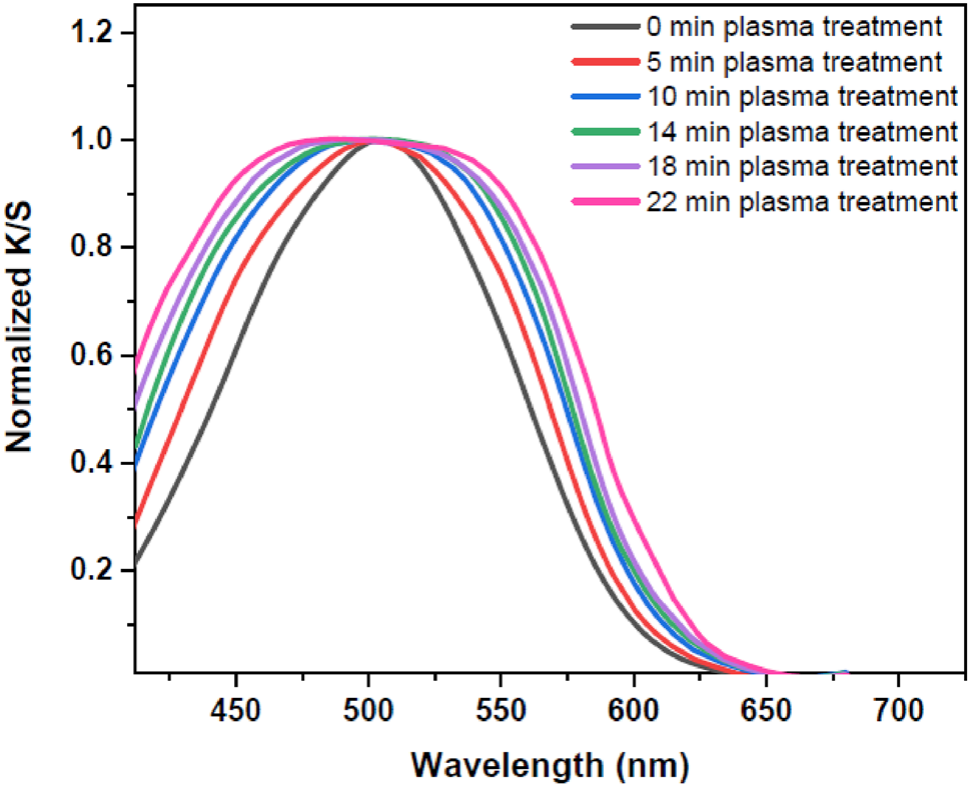
Normalized K/S for the washed untreated and plasma-treated dye-loaded samples for different time of plasma modification.

**Figure 9:**
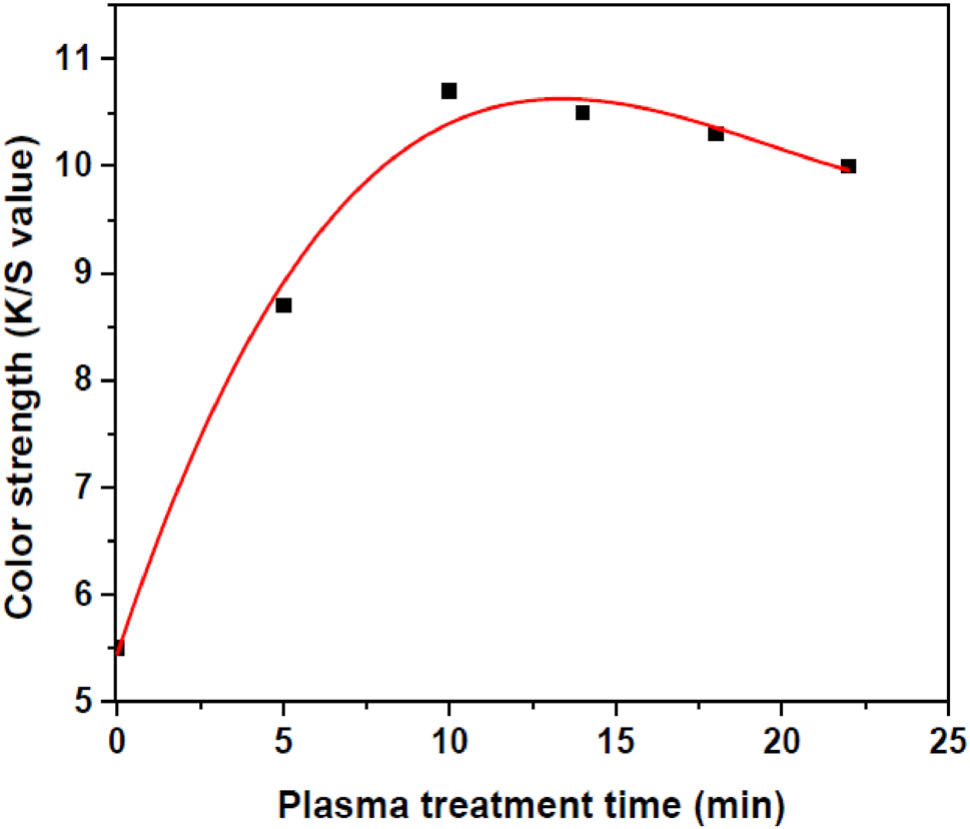
Effect of plasma on the color strength of DR1 dye-loaded polyamide 6 polymer.

K/S values of the untreated and plasma-treated dye-loaded samples with different treatment time were obtained after DMF extraction for 40 min. Table 4 shows the effect of plasma on K/S values of the samples. The color strength (K/S values) changes of the polymeric films with increasing plasma treatment time is shown in Fig. 8. Also, Fig. 9 and Table 5 shows the changes of wavelength for maximum K/S value.

**Table 4:** K/S values of the dye-loaded samples after DMF extraction for 40 min.

Samples	K/S values after DMF extraction
Untreated	5.51
5 min plasma-treated	8.73
10 min plasma-treated	10.75
14 min plasma-treated	10.52
18 min plasma-treated	10.33
22 min plasma-treated	10.19

**Table 5:** The wavelength of maximum K/S for the washed untreated and plasma treated samples at different time of modification.

Samples	λ_max_
Untreated	503.5
5 min plasma-treated	501.1
10 min plasma-treated	498.2
14 min plasma-treated	497.4
18 min plasma-treated	496.7
22 min plasma-treated	495.9

## Discussion

As shown in Fig. 1, the comparison between the FT-IR spectra of the pristine and plasma-treated samples indicates that the intensity of the tertiary amide peak is noticeably increased by plasma treatment, which demonstrates that the plasma can convert the primary and secondary amide groups in the surface composition of the polymer to the tertiary amides. Also, the indentation at the hillside in the region of 3200–3400 cm^−1^ confirms a decrease in N–H bonds due to plasma modification.

As a result, argon gas plasma treatment forms radical sites on the surface of polyamide 6 polymer. The active sites lead to surface activation by hydrogen abstraction of the polymer surface. According to the literature, hydroxyl groups can be separated from DR1 dye under similar conditions^49^. Also, in the range of 1000 to 1300 cm^−1^, the intensity of the peak related to C-N bond increases. The active surface sites of polyamide 6 films interact with the dye, and as a result, the hydroxyl part of the dye gives a compression reaction with the amine polymer group. The dye reactions with the active radical sites on the polymer, created by the plasma, are illustrated in Fig. 8.

**Figure 10:**
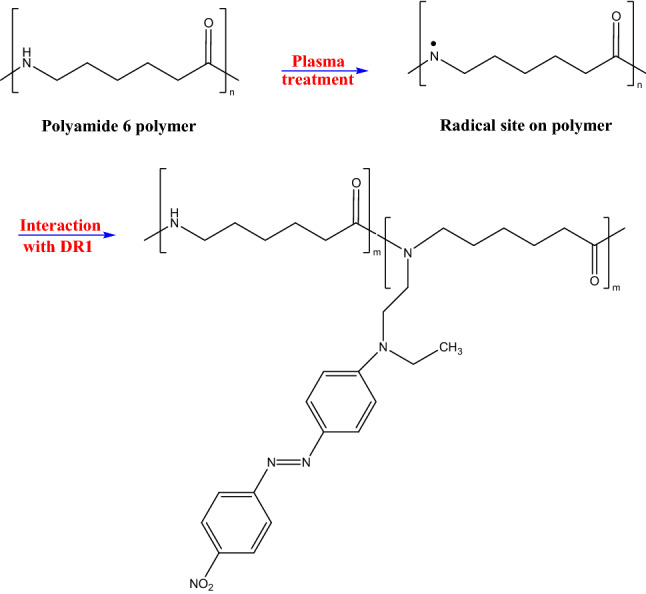
Schematic illustration for chemical changes of nylon 6 doped with DR1 dye structure due to plasma treatment.

As shown in Fig. 1, the FT-IR analysis of the dye-doped polymer sample shows that the presence of DR1 dye in polyamide 6 polymer layer intensifies the creation of tertiary amides. The obtained results are in full compliance with the mechanism presented in Fig. 8. Finally, the formation of covalent bonds causes the dye to become immobilized on the polymer surface (Fig. 10).

The increase in the peak intensity of tertiary amides in the doped polymer state is more than pure polymer film. In addition, the peak related to 1635 cm^−1^ is broadened, and a new bulge is seen around 1733 cm^−1^ to the right of the peak in the treated dye-doped sample. In other words, the plasma also induces new carbonyl groups on the surface of the polyamide 6 polymer using components segregated from the surface material. Furthermore, the plasma-activated surface of the polymer can react with the oxygen in the air to form such groups after being exposed to ambient air. Creating new C=O and C–O species raises the polarity of the polymer surface and increases the surface free energy and wettability, leading to more dye adsorption into the polymer surface^51^. For example, creation of some components such as carbonyl, hydroxyl, and carboxylic acid makes the surface more active^27,52^. As a result, substances called low molecular weight oxidized material (LMWOM) are formed on the surface of the polymer after exposure to ambient air that increase the amount of some polar materials on the surface, like C=O and C–O bonds^53^, which act as an intermediary to adsorb the dye into the polymer surface. Oxygenated compounds can help the dye adhere to the polymer surface by creating a hydrogen bond.

According to Fig. 2, there are two possibilities to explain the changes of UV–Vis spectra of the sample due to different times of plasma treatment. One possibility is attributed to dye spatial conformational changes. The other is related to plasma etching and sputtering of the polymer surface, which reduces the intensity of the resulting spectra obtained by UV–Vis spectra. In the following, two possibilities are examined in more detail.

The absorption spectra obtained from the DR1 dye-doped into polyamide 6 polymer film, before and after plasma treatment for different times, were used to determine the alteration of dye conformation due to argon plasma treatment. Azobenzene dyes have two main peaks for each conformation in the UV–Vis spectra arising from π → π* and n → π* transitions. The intensity of absorption band of π → π* transition in the trans species is higher than the cis states, but the π → π* and n → π* transitions are slightly shifted further in the wavelength range for the cis mode^37,54^. The introduced peaks in the UV–Vis absorption spectrum of the DR1 dye, as a pseudo-stilbene type dye, contributing in both isomeric states of this type of dyes, are so close to each other and overlapped^55^. The absorption spectra can be used to estimate the cis to trans isomers ratio before and after plasma treatment through calculation of the area under the deconvoluted curves. In this work, the absorption spectra were fitted by two Gaussian peaks to extract and compare the amount of cis and trans isomeric species using the OriginPro software. Figure 3 shows the deconvoluted DR1 dye-doped polyamide 6 polymer films' absorption spectra before and after plasma treatment.

The area under the dependent fitted curves in the absorption spectra, due to plasma treatment in different time intervals, confirms an increase in the population of cis isomeric species compared to trans types, see Table 1. It seems that the DR1 azo dye doped in the polymer shows trans–cis photo-isomerization by absorbing energy from the plasma medium. This photo-isomerization state occurs in a polymeric environment, and so the polymeric environment around the dye can be re-oriented. Therefore, the return to the stable trans form is disturbed in the dark, so the samples do not show the conversion of the cis form to the trans form after a long time (about a month).

As shown in Table 2, the roughness parameters of the polymeric surface have developed after argon plasma treatment (300 s modification) compared to the control sample. The amount of R_a_ roughness as the average deviation from the surface baseline is obviously enhanced. Also, AFM results show that the R_q_ roughness are increased after glow discharge plasma treatment of the dye-doped polymer surface. According to Fig. 4, the plasma creates pores on the surface of the polymer, and spikes of the polymer surface increase the roughness on the polyamide 6 surface as a result of etching and sputtering by plasma. In addition to etching and sputtering, some compounds can adhere to the polymer surface during or after the plasma treatment and change the surface roughness.

According to Fig. 5, increasing the plasma treatment time reduces the intensity of the resulting spectra obtained by UV–Vis spectra of DR1 dye-doped polymer film with extra surface dye. It should be noted that this reduction in intensity is more significant than the DR1 dye-doped polyamide 6 polymer film without extra dye on surface, which was discussed formerly. As can be seen from Fig. 6, the absorption spectra of the sample before and after different plasma treatment times, were deconvoluted to investigate the relative population of cis to trans isomers. A comparison between the acquired results (Table 3) indicates that the plasma affects the surface of materials more than the bulk. Also, the cis isomer has a higher ability to form and stabilize in the case of extra dye loading on the surface of the polymer. Also, the samples remained stable for about a month in a dark environment. The conversion rate of the cis to trans form depends on the empty space surrounding the dye^56^. Obviously, there are more empty spaces for dye isomerization on the surface of the polymer than the bulk. It is clear that more effective photons in the plasma environment collide with the dye molecules on the surface directly. The plasma changes the conformation of many dye molecules on the surface due to more free space than dye molecules in the bulk of the material. Some of the reasons for the stabilization of the cis conformation are because of locking the cis isomer due to dye alignment and spatial limitation^57^. On the other hand, plasma can change surface morphology and chemistry, and prepare the surface conditions to stable cis isomers physically. Increasing the number of pores and polar groups on the surface of the polymer by plasma contributes to stabilizing the dye in the cis state. Numerous subjects impress on the dye inversion, such as substituent, environmental polarity and temperature, and the other environmental conditions^57,58^. Creating new bonds between the dye and the polymer and new substituent of dye affect the stability of the cis isomer state. Plasma also affects the substituent of the dye and alters various chemical and physical characteristics of the surface. By creating bonds between the dye and the polymer, the plasma causes changes in the optical behavior and stability of the dye, in particular isomeric states. Additionally, in this study, the DC field applied for plasma creates some components with partial dipoles oriented in the field’s direction on the surface. This leads to a regular polarization that brings about alignment, stability, and adhesion of the dye to the surface of the polymer, which is helpful for various nonlinear optical applications. In addition, the higher energy level of the cis isomer leads to more significant reactions with the polymer surface and causes the dye to become immobilized on the polymer surface. Therefore, it is obvious that the plasma increases the adhesion and durability of the dye on the surface. Polymers have good surface and optical properties, and it is easy to make thin films of polymeric material. Even though inorganic materials are usually used to store information due to greater stability of isomeric state of the dye^41^, plasma treatment suggests a new approach for stabilizing the cis isomer by a strong influence on the polymer surface and dye to create a better polymer environment for data storage and optical applications.

By comparing the UV–Vis spectra of Fig. 7 of the untreated and plasma-treated samples after aging process, it is concluded that plasma causes the dye to adhere more to the polymer surface than the sample without plasma treatment. Plasma modification of the dye-loaded surface of polymer significantly increases the number of dye molecules attaching to the surface and diminishes discoloration of the polymer during the aging process. The tabulated information of Table 4 the fitted curve of Fig. 8 demonstrate that plasma increases the dyeability of the surface of polyamide 6 polymer. The color strength of the samples after washing process increases with the increase of plasma treatment time, and 10 min argon plasma treatment time can be the optimized modification time for enhancement of color strength with these experimental conditions. The results show that plasma can create strong links between the dye and polymer than increases dye durability on the surface of polymer.

## Conclusions

The effects of argon DC glow discharge plasma on dyeing of polyamide 6 polymer surface were investigated. The results confirm that plasma-modified dye-loaded film suffers less dye leaching because of physical and chemical changes of the surface content. Plasma activates the surface of polyamide 6 polymer and creates radical groups through breaking some specific bonds on the surface. The subsequent reactions create covalent bonds leading to the increase of tertiary amides and immobilization of dye on the surface of polymer. Besides, plasma treatment of polymeric films increases electro-negativity and surface polarity by introducing polar groups that reinforce the dye’s durability on the polymer surface. Plasma process introduces a new and effective method for converting trans to cis isomer states of DR1 dye with a higher conversion rate in nylon 6 polymer film. The higher energy of cis species enhances the reactivity of the dye with the polymer surface and increases the possibility of dye reactions with the surface. Plasma has significant effect on the conformational conversion and stability of the dye molecules present on the surface because of changes in substituent and polarity of the surface. The results demonstrate that the glow discharge plasma increases the interactive surface of the polymer by enhancing roughness and creating fine and porous structures on the surface, which improves the adhesion of the dye to the surface of the polymer. On the other hand, the surface roughness positively affects the stability of the cis isomer of the dye during plasma modification.

Finally, plasma modification of the polymer surface can be an appropriate technique for dye immobilization on the surface. This technique increases color fastness on the surface and prepares it for use in different applicable substances like halochromic and holographic materials. Moreover, using plasma can multiply the capacity of dye-based optical memories or data storage systems and increase their stability. Also, increasing the amount of cis isomers of dye on the polymeric surface prevents degradation of the polymer through UV radiation absorption on the surface.

## Materials and methods

### Materials

Polyamide 6 polymer, with the linear formula of [–NH(CH_2_)_5_CO–]_n_, used in this work, was purchased from Sigma-Aldrich with a density of 1.084 g/mL at the temperature of 25 °C and the glass transition temperature of 47 °C. DR1 dye, formic acid (HCOOH), and ethanol (C_2_H_6_O) as solvents and dimethylformamide (DMF) were obtained from Merck. The water used in this work was distilled water.

### Characterization instruments

70, an FT-IR spectrophotometer, was used to record the vibration spectra over the wavenumber range of 400–4000 cm^−1^ with the accuracy of 3 cm^−1^. A double beam Shimadzu UV-2450 UV–Visible spectrophotometer was used to record the absorption spectra over a wavelength range of 200–900 nm. Spectroscopic instruments were combined with a cell temperature controller with an accuracy of ± 0.1 °C. Also, the reflectance percentage of the samples were recorded by this instrument. The atomic force microscope (AFM) analysis was performed on a Nanosurf Mobile S instrument.

### Aging test

An accelerated aging process was chosen to examine the immobilization of the dye on the surface of the polymer. The color fastness was compared between the plasma-treated and untreated dye-loaded polymer films during the aging test. The films were placed in an aging oven (chamber with quartz walls) exposed to an ultraviolet light at the temperature of 45 °C and humidity of 87%. The ultraviolet light source was a 500 W mercury-vapor lamp. It was used for various time intervals at a distance of 22 cm from all samples’ surfaces at equal angles, and humidity of 87% was obtained by evaporating distilled water from the floor of the chamber. After the aging process at different times, the UV–Vis absorption spectra were recorded to determine the color retention on the surface of the samples.

### Sample preparation

The polymer solution was prepared by dissolving polyamide 6 polymer in formic acid at a concentration of 0.5 W/W% at room temperature. The nylon 6 films were cast on glass slides by the spin-coating method. Generally, three sets of samples were prepared; the first set includes pure polyamide 6 polymer films to investigate the chemical changes and the amount of dye fastness on the surface of pure polymer films immersed in dye solution before and after plasma treatment. The second set of films was cast from the polyamide 6 solution doped with DR1 dye at a concentration of 2 W/W% to study physical and chemical changes of surface content. For the last group, the prepared doped polymer films were dipped in the solution of DR1 dye in ethanol solvent with a concentration of 10 mg/mL in ambient temperature for optimized time of 90 s, so the films adsorb more dye homogeneously. These samples were used for conformational studies. It should be noted that the prepared samples were dried at a temperature of 30 °C in an oven. The films were exposed to a plasma environment, and the surface changes were compared with the plasma-free state. The experiments were repeated three times for each batch of samples.

### Plasma instrument

The chamber of glow discharge plasma in this work was a Pyrex tube with a length of 500 mm with two aluminum electrodes attached to the two ends of this cylindrical tube. The polymer films were placed in the positive column zone of the glow discharge plasma for various time durations. The applied gas was argon (with a purity of 99.99%), and the gas pressure in the plasma chamber was decreased with a rotary vacuum pump (Alcatel) joint. After purging the chamber with argon gas, the target gas pressure was fixed at 2 × 10^–1^ Torr. Then the power supply connected to the electrodes introduced a glow discharge plasma in the space between the two electrodes by applying the DC bias voltage of 1.1 kV and discharge current of 0.15 A with the power density of 660 mW/cm^3^. After plasma modification of the samples, the chamber pressure reaches the atmospheric pressure. Figures S4 and S5 in supplementary information indicate respectively the image and scheme of argon DC glow discharge plasma instrument treating a sample.

### Statistical analysis

The absorption spectra were fitted by two Gaussian peaks to extract and compare the amount of cis and trans isomeric species using the OriginPro 2018 software. The area under each deconvoluted curve indicates the population of any isomeric state. The raw data of UV–Vis spectra were smoothed by the OriginPro software using Savitzky-Golay method with 32 points of window.

### Determination of color strength

A batch of dye-doped samples immersed in dye solution, were washed with aqueous 50% DMF at the temperature of 45 °C for 40 min after plasma treatment and compared with untreated washed samples with the same conditions. The color strength (K/S value) of each sample was determined by reflectance measurement according to Kubelka–Munk equation^59^.

## Supplementary Information

Supplementary Information.

## Data Availability

The datasets obtained during the current study are available from the corresponding authors on reasonable request.

## References

[1] Koo, J. H. *Polymer Nanocomposites: Processing, Characterization, and Applications*. (McGraw-Hill Education, 2019).

[2] Feldman D (2017). Polyamide nanocomposites. J. Macromol. Sci. Part A Pure Appl. Chem..

[3] Nair SS, Ramesh C (2005). Studies on the crystallization behavior of nylon-6 in the presence of layered silicates using variable temperature WAXS and FTIR. Macromolecules.

[4] Peng X (2019). Shape memory effect of three-dimensional printed products based on polypropylene/nylon 6 alloy. J. Mater. Sci..

[5] Yanilmaz M, Dirican M, Zhang X (2014). Evaluation of electrospun SiO_2_/nylon 6,6 nanofiber membranes as a thermally-stable separator for lithium-ion batteries. Electrochim. Acta.

[6] Ding R, Bowler N (2015). Permittivity and electrical breakdown response of nylon 6 to chemical exposure. IEEE Trans. Dielectr. Electr. Insul..

[7] Mei Z, Chung DDL (2001). Thermal history of carbon-fiber polymer-matrix composite, evaluated by electrical resistance measurement. Thermochim. Acta.

[8] Sahu SK, Mahto RP, Pal K (2020). Investigation on mechanical behavior of friction stir welded nylon-6 using temperature signatures. J. Mater. Eng. Perform..

[9] Savarino P (2000). Effects of additives on the dyeing of nylon-6 with dyes containing hydrophobic and hydrophilic moieties. Dye. Pigment..

[10] Awaja F, Gilbert M, Kelly G, Fox B, Pigram PJ (2009). Adhesion of polymers. Prog. Polym. Sci..

[11] Ihalainen P (2012). Influence of surface properties of coated papers on printed electronics. Ind. Eng. Chem. Res..

[12] Amirilargani M, Sadrzadeh M, Sudhölter EJR, de Smet LCPM (2016). Surface modification methods of organic solvent nanofiltration membranes. Chem. Eng. J..

[13] Shin J, Liu X, Chikthimmah N, Lee YS (2016). Polymer surface modification using UV treatment for attachment of natamycin and the potential applications for conventional food cling wrap (LDPE). Appl. Surf. Sci..

[14] Ayrilmis N, Jarusombuti S, Fueangvivat V, Bauchongkol P (2011). Effect of thermal-treatment of wood fibres on properties of flat-pressed wood plastic composites. Polym. Degrad. Stab..

[15] Maroofi A, Navab Safa N, Ghomi H (2020). Atmospheric air plasma jet for improvement of paint adhesion to aluminium surface in industrial applications. Int. J. Adhes. Adhes..

[16] Xiao X (2018). Argon plasma treatment to tune perovskite surface composition for high efficiency solar cells and fast photodetectors. Adv. Mater..

[17] Bergmann JB (2020). Polymerization-induced wrinkled surfaces with controlled topography as slippery surfaces for colorado potato beetles. Adv. Mater. Interfaces.

[18] Pankaj SK, Wan Z, Keener KM (2018). Effects of cold plasma on food quality: A review. Foods.

[19] Wei X (2012). CF 4 plasma surface modification of asymmetric hydrophilic polyethersulfone membranes for direct contact membrane distillation. J. Memb. Sci..

[20] Jaleh B, Parvin P, Wanichapichart P, Saffar AP, Reyhani A (2010). Induced super hydrophilicity due to surface modification of polypropylene membrane treated by O_2_ plasma. Appl. Surf. Sci..

[21] Tušek L, Nitschke M, Werner C, Stana-Kleinschek K, Ribitsch V (2001). Surface characterisation of NH_3_ plasma treated polyamide 6 foils. Colloids Surf. A.

[22] de Groot GJJB, Hundt A, Murphy AB, Bange MP, Mai-Prochnow A (2018). Cold plasma treatment for cotton seed germination improvement. Sci. Rep..

[23] France RM, Short RD (1998). Plasma treatment of polymers: The effects of energy transfer from an argon plasma on the surface chemistry of polystyrene, and polypropylene. A high-energy resolution X-ray photoelectron spectroscopy study. Langmuir.

[24] Attri P, Arora B, Choi EH (2017). Retraction: Utility of plasma: A new road from physics to chemistry. RSC Adv..

[25] Owen MJ, Stasser JL (1997). Plasma treatment of polydimethylsiloxane. Am. Chem. Soc. Polym. Prepr. Div. Polym. Chem..

[26] Randeniya LK, De Groot GJJB (2015). Non-thermal plasma treatment of agricultural seeds for stimulation of germination, removal of surface contamination and other benefits: A review. Plasma Process. Polym..

[27] Kostov KG, Nishime TMC, Castro AHR, Toth A, Hein LRO (2014). Surface modification of polymeric materials by cold atmospheric plasma jet. Appl. Surf. Sci..

[28] Shaw D, West A, Bredin J, Wagenaars E (2016). Mechanisms behind surface modification of polypropylene film using an atmospheric-pressure plasma jet. Plasma Sources Sci. Technol..

[29] Chan CM, Ko TM, Hiraoka H (1996). Polymer surface modification by plasmas and photons. Surf. Sci. Rep..

[30] De Smet L (2018). Plasma dye coating as straightforward and widely applicable procedure for dye immobilization on polymeric materials. Nat. Commun..

[31] Wong JKH, Tan HK, Lau SY, Yap PS, Danquah MK (2019). Potential and challenges of enzyme incorporated nanotechnology in dye wastewater treatment: A review. J. Environ. Chem. Eng..

[32] Roy U (2018). Assessment on the decolourization of textile dye (reactive yellow) using Pseudomonas sp. immobilized on fly ash: Response surface methodology optimization and toxicity evaluation. J. Environ. Manage..

[33] Priimagi A, Kaivola M, Virkki M, Rodríguez FJ, Kauranen M (2010). Suppression of chromophore aggregation in amorphous polymeric materials: Towards more efficient photoresponsive behavior. J. Nonlinear Opt. Phys. Mater..

[34] Yager KG, Barrett CJ (2006). Novel photo-switching using azobenzene functional materials. J. Photochem. Photobiol. A Chem..

[35] Merino E, Ribagorda M (2012). Control over molecular motion using the cis-trans photoisomerization of the azo group. Beilstein J. Org. Chem..

[36] Nançoz C (2018). Influence of the hydrogen-bond interactions on the excited-state dynamics of a push-pull azobenzene dye: The case of Methyl Orange. Phys. Chem. Chem. Phys..

[37] Cho EN, Zhitomirsky D, Han GGD, Liu Y, Grossman JC (2017). Molecularly engineered azobenzene derivatives for high energy density solid-state solar thermal fuels. ACS Appl. Mater. Interfaces.

[38] Al-Bataineh QM (2020). Kinematics of photoisomerization processes of PMMA-BDK-MR polymer composite thin films. Polymers (Basel)..

[39] Han GGD, Li H, Grossman JC (2017). Optically-controlled long-term storage and release of thermal energy in phase-change materials. Nat. Commun..

[40] Steyaert I, Vancoillie G, Hoogenboom R, De Clerck K (2015). Dye immobilization in halochromic nanofibers through blend electrospinning of a dye-containing copolymer and polyamide-6. Polym. Chem..

[41] Gao T, Xue Y, Zhang Z, Que W (2018). Multi-wavelength optical data processing and recording based on azo-dyes doped organic-inorganic hybrid film. Opt. Express.

[42] Samai S, Bradley DJ, Choi TLY, Yan Y, Ginger DS (2017). Temperature-dependent photoisomerization quantum yields for azobenzene-modified DNA. J. Phys. Chem. C.

[43] Deloncle R, Caminade AM (2010). Stimuli-responsive dendritic structures: The case of light-driven azobenzene-containing dendrimers and dendrons. J. Photochem. Photobiol. C Photochem. Rev..

[44] Zakerhamidi MS (2010). Isotropic and anisotropic environment effects on the UV/vis absorption spectra of three disperse azo dyes. J. Mol. Liq..

[45] Zibaei R, Zakerhamidi MS, Korram S, Ranjkesh A (2021). Effects of polarized light on the optical and self-oscillation behaviors of liquid crystal network polymers. J. Mater. Chem. C.

[46] Seyednoruziyan B (2021). Improving the optoelectronic efficiency of novel meta-azo dye-sensitized TiO_2_ semiconductor for DSSCs. Spectrochim. Acta. Part A Mol. Biomol. Spectrosc..

[47] Aamoum A (2020). Time-resolved photoluminescence and optical properties of a specific organic azo dye. Opt. Quantum Electron..

[48] Pavia, D. L., Lampman, G. M. & Kriz, G. S. *Introduction to Spectroscopy* 3rd edn. (Thomson Learning Inc., 2001).

[49] Siow KS, Britcher L, Kumar S, Griesser HJ (2006). Plasma methods for the generation of chemically reactive surfaces for biomolecule immobilization and cell colonization: A review. Plasma Process. Polym..

[50] Hosseini S, Ibrahim F, Djordjevic I, Koole LH (2014). Recent advances in surface functionalization techniques on polymethacrylate materials for optical biosensor applications. Analyst.

[51] Švorčík V (2006). Modification of surface properties of high and low density polyethylene by Ar plasma discharge. Polym. Degrad. Stab..

[52] Moghadamzadeh H, Rahimi H, Asadollahzadeh M, Hemmati AR (2011). Surface treatment of wood polymer composites for adhesive bonding. Int. J. Adhes. Adhes..

[53] Bagiatis V, Critchlow GW, Price D, Wang S (2019). The effect of atmospheric pressure plasma treatment (APPT) on the adhesive bonding of poly(methyl methacrylate) (PMMA)-to-glass using a polydimethylsiloxane (PDMS)-based adhesive. Int. J. Adhes. Adhes..

[54] Das D (2020). Understanding of the kinetic stability of cis- isomer of azobenzenes through kinetic and computational studies. ChemistrySelect.

[55] Mahimwalla Z (2012). Azobenzene photomechanics: Prospects and potential applications. Polym. Bull..

[56] Mitus AC, Pawlik G, Kajzar F, Grote JG (2008). Kinetic Monte Carlo study of diffraction grating recording/erasure in DNA-based azo-dye systems. Nanobiosyst. Process. Charact. Appl..

[57] Yager KG, Barrett CJ (2008). Azobenzene polymers for photonic applications. Smart Light. Mater. Azobenzene-Contain. Polym. Liq. Cryst..

[58] Wazzan NA, Richardson PR, Jones AC (2010). Cis-trans isomerisation of azobenzenes studied by laser-coupled NMR spectroscopy and DFT calculations. Photochem. Photobiol. Sci..

[59] Christy AA, Kvalheim OM, Velapoldi RA (1995). Quantitative analysis in diffuse reflectance spectrometry: A modified Kubelka–Munk equation. Vib. Spectrosc..

