# Supplementary material for: Plasma immobilization of azobenzene dye on polyamide 6 polymer
Source: Sci Rep. 2023 Jan 18;13:983. doi: 10.1038/s41598-023-27484-9 (PMC9849228; doi:10.1038/s41598-023-27484-9)
Supplement: Supplementary file 1 — Supplementary Information. [file 41598_2023_27484_MOESM1_ESM.docx]

Supplementary Information

**Plasma Immobilization of Azobenzene Dye on Polyamide 6 Polymer**

**Mohammad Reza Yari^1,^*^,+^,** **Mohammad Sadegh Zakerhamidi^2,3,4^,** **and Hamid Ghomi^1,^*^,+^,**

^1^ Laser and Plasma Research Institute, Shahid Beheshti University, Tehran, Iran.

^2^ Faculty of Physics, University of Tabriz, Tabriz, Iran.

^3^ Research Institute for Applied Physics and Astronomy, University of Tabriz, Tabriz, Iran.

^4^ Photonics Center of Excellence, University of Tabriz, Tabriz, Iran.

* Corresponding authors

Mohammad Reza Yari, E-mail: [M_Yari@sbu.ac.ir](mailto:M_Yari@sbu.ac.ir)

Hamid Ghomi, E-mail: [h-gmdashty@sbu.ac.ir](mailto:h-gmdashty@sbu.ac.ir)

^+^ These authors contributed equally to this work.


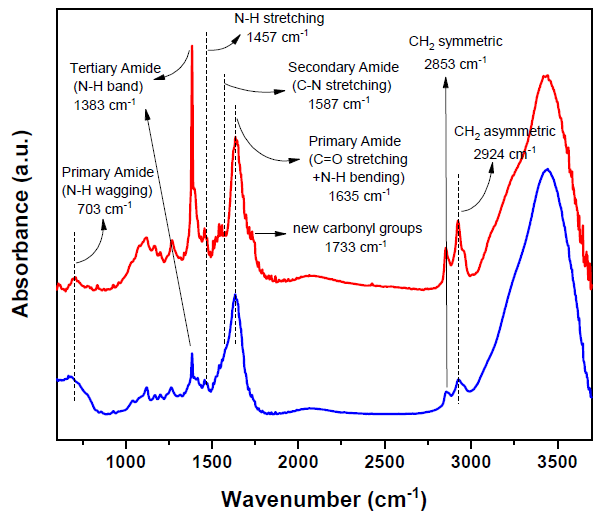


**Figure S1.** The detailed peaks information of FT-IR spectra of DR1 dye-doped polyamide 6 polymer (blue line) untreated sample (red line) argon plasma-treated sample for 300 s modification


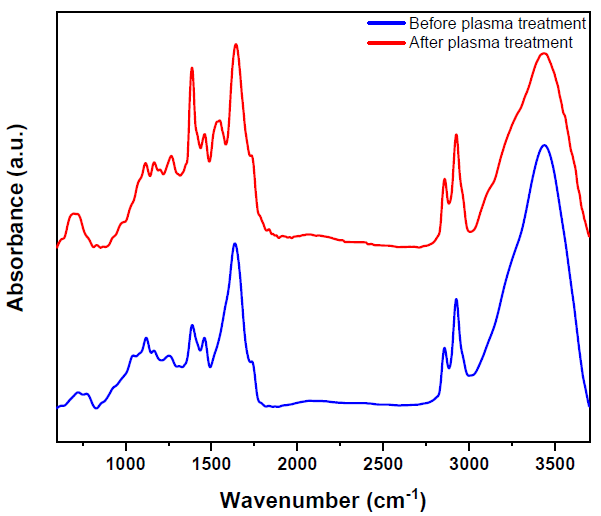

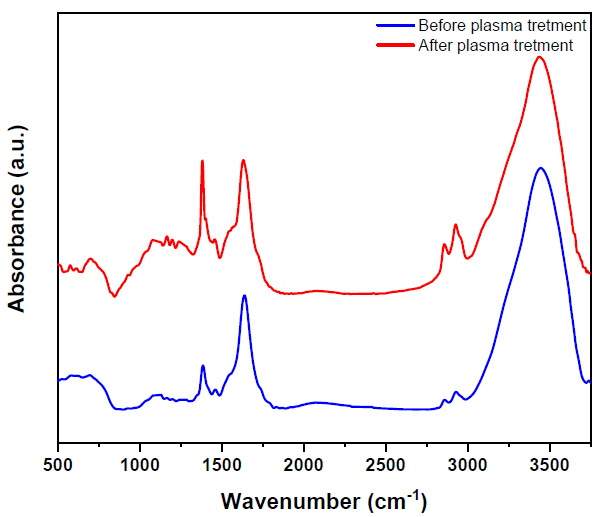


**Figure S2.** FT-IR spectra of (left) pure polyamide 6 film (right) DR1 dye-doped polyamide 6 polymer (blue lines) untreated sample (red lines) argon plasma-treated sample for 150 s modification


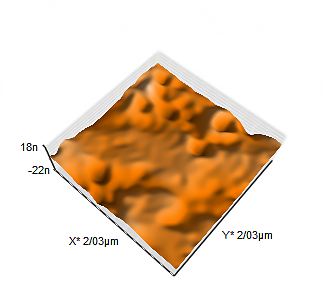

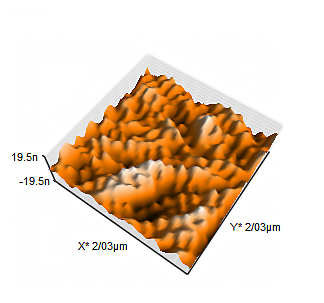


**Figure S3.** AFM images of (left) untreated nylon 6 surface (right) argon plasma-treated polyamide 6 surface for 150 seconds modification


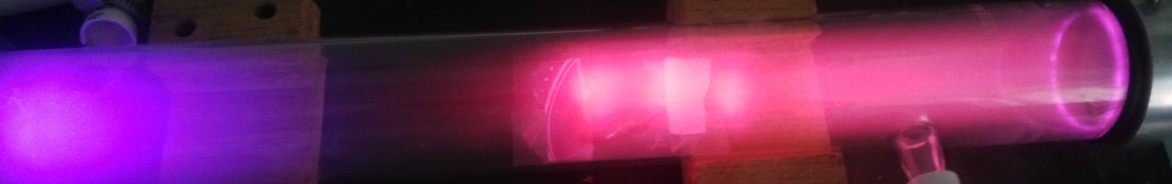


**Figure S4.** Image of argon DC glow discharge plasma


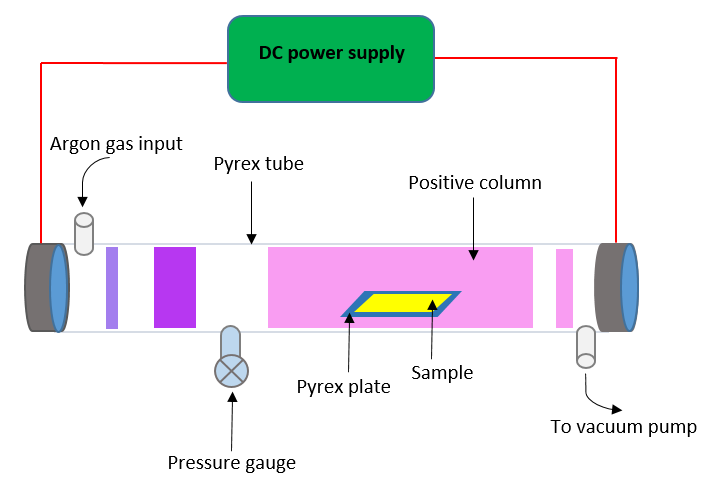


**Figure S5.** Schematic figure showing glow discharge plasma system
